# Differential Expression of MicroRNAs in CD34+ Cells of 5q- Syndrome

**DOI:** 10.1186/1756-8722-4-1

**Published:** 2011-01-06

**Authors:** Hana Votavova, Martina Grmanova, Michaela Dostalova Merkerova, Monika Belickova, Alzbeta Vasikova, Radana Neuwirtova, Jaroslav Cermak

**Affiliations:** 1 Department of Molecular Genetics, Institute of Hematology and Blood Transfusion, Prague, Czech Republic; 2 1st Medical Department of Charles University Hospital, Prague, Czech Republic; 3 Institute of Clinical and Experimental Hematology, 1st Faculty of Medicine, Charles University, Prague, Czech Republic

## Abstract

**Background:**

Myelodysplastic syndrome with isolated chromosome 5q deletion (5q- syndrome) is a clonal stem cell disorder characterized by ineffective hematopoiesis. MicroRNAs (miRNAs) are important regulators of hematopoiesis and their aberrant expression was detected in some clonal hematopoietic disorders. We thus analyzed miRNA expressions in bone marrow CD34+ cells of 5q- syndrome patients. Further, we studied gene expressions of *miR-143*, *miR-145*, *miR-378 *and *miR-146a *mapped within the 5q deletion.

**Results:**

Using microarrays we identified 21 differently expressed miRNAs in 5q- patients compared to controls. Especially, *miR-34a *was markedly overexpressed in 5q- patients, suggesting its role in an increased apoptosis of bone marrow progenitors. Out of four miRNAs at del(5q), only *miR-378 *and *miR-146a *showed reduced gene expression in the patients. An integrative analysis of mRNA profiles and predicted putative targets defined potential downstream targets of the deregulated miRNAs. The list of targets included several genes that play an important role in the regulation of hematopoiesis (e.g. *KLF4*, *LEF1*, *SPI1*).

**Conclusions:**

The study demonstrates global overexpression of miRNAs is associated with 5q- phenotype. Identification of hematopoiesis-relevant target genes indicates that the deregulated miRNAs may be involved in the pathogenesis of 5q- syndrome by a modulation of these targets. The expression data on miRNAs at del(5q) suggest the presence of mechanisms for compensation of a gene dosage.

## Background

The 5q- syndrome is a distinct subtype of myelodysplastic syndrome (MDS) with typical molecular, cytogenetic, morphological, clinical and prognostic features; an isolated interstitial deletion of the long arm of chromosome 5 [del(5q)], bone marrow blasts less than 5%, normal or often increased platelet count, macrocytic anemia, typical megakaryocytes and often hypoplastic erythropoiesis. Pathophysiological basis of 5q- syndrome is likely associated with haploinsufficiency of genes mapping to the deleted region at 5q31-q32, so called commonly deleted region (CDR). However, other mechanisms may contribute to the ineffective hematopoiesis in 5q- syndrome.

MicroRNAs (miRNAs) are small non-coding RNAs that negatively modulate expression of complementary genes by translation inhibition or mRNA degradation. MiRNAs have been shown to be important regulators of hematopoiesis and their aberrant expression was found in some clonal hematopoietic disorders such as polycythemia vera [1,2]. In MDS, Pons et al. analyzed gene expression of 25 hematopoiesis-relevant miRNAs in mononuclear cells and examined possible association of their expression with other parameters such as disease stage, risk score etc. [3]. Hussein et al. performed miRNA expression profiling in total bone marrow (BM) cells of MDS patients with normal karyotype and distinct cytogenetic aberrations [4]. However, there is limited information on miRNA regulation in BM progenitors of MDS. Thus, we determined miRNA expression patterns in BM CD34+ cells of 5q- syndrome patients and searched for differentially expressed miRNAs that might contribute to the pathogenesis of 5q- syndrome. To define potential downstream targets of the deregulated miRNAs in 5q- patients, we combined mRNA microarray data of the tested patients with those of *in silico *miRNA target predictions. Further, we attempted to address a gene dosage effect of del(5q) on gene expression of the miRNAs mapped within the deletion in BM progenitors and peripheral blood cells of 5q- patients.

## Results and Discussion

Ineffective hematopoiesis, the hallmark of MDS, arises from defective hematopoietic progenitors that display retarded maturation capacity, premature apoptotic death, and impaired growth and responsiveness to growth factors. However, recent studies of miRNAs in MDS were performed on partly separated or non-separated cells of bone marrow [3,4]. In this study, we thus focused on progenitor cells of 5q- syndrome in order to determine miRNA expressions specific for this cell population.

**Figure 1:**
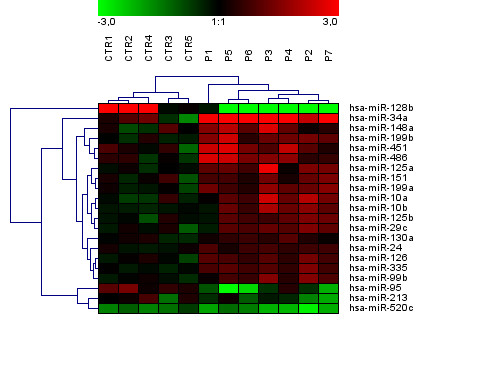
**Unsupervised hierarchical clustering of 21 differentially expressed miRNAs between controls and 5q- patients (p < 0.05 after Bonferroni correction)**. The relative miRNA expression changes are expressed by a color gradient intensity scale, as shown at the top. The lightest green color indicates maximal decrease and the lightest red color indicates maximal increase of gene expression. Each column represents a separate CD34+ sample and each row a single miRNA. CTR- control, P- patient.

MiRNA expression profiles were assayed in bone marrow (BM) CD34+ cells from 5q- syndrome patients and controls using TaqMan arrays with 365 probes. Out of the miRNA set, transcripts of 183 miRNAs at average were expressed at the detectable level (C_T_< 35). Comparative analysis determined differential expression of 21 miRNAs between 5q- patients and controls at p < 0.05 after Bonferroni correction; increased expression of 17 miRNAs and decreased expression of 4 miRNAs in 5q- patients. Unsupervised hierarchical clustering performed using this miRNA set clearly discriminated 5q- patients from controls [Figure 1]. Higher proportion of up-regulated miRNAs inversely correlated with global down-regulation of mRNA expressions in MDS reported previously [5]. The averaged fold changes of miRNA expressions in 5q- patients were summarized in the Additional file 1.

Pons et al. analyzed expression of 25 hematopoietic miRNAs in BM mononuclear cells of MDS patients and identified 12 overexpressed miRNAs including *miR-10a*, *miR-10b *and *miR-126 *[3]. In our study, we also detected up-regulation of these miRNAs in 5q- progenitors. *miR-10a *and *miR-10b *genes are embedded in the cluster of *HOX *genes that are implicated in early hematopoiesis as well as leukemogenesis. Expression of these miRNAs correlates with *HOX *gene expression, suggesting their modulation by the same regulators as those of *HOX *genes. For example, *miR-126 *is co-expressed with *HOXA9 *mRNA in hematopoietic stem cells and down-regulated in parallel during progenitor differentiation [6]. Overexpression of *HOXA9 *was observed in BM samples of unselected MDS patients [7] and it caused stem cell expansion in BM mouse cells [8]. It suggests that up-regulation of *miR-126 *may be associated with the clonal cell expansion in 5q- syndrome. *miR-99b *and *miR-130a*, up-regulated in our 5q- patients, represented other miRNAs involved in the regulation of *HOX *genes.

*miR-34a *showed the most up-regulated expression in 5q- patients compared to controls (12-fold at average, p < 0.01). This miRNA is a direct proapoptotic transcriptional target of *p53 *that in turn regulates the expression of some *p53 *target genes. Induced expression of *miR-34a *activates apoptosis by inhibition of *BCL2 *target gene [9]. Thus, high expression of *miR-34a *in 5q- patients is likely to be related to an increased apoptosis of BM progenitors. On the other hand, decreased expression of *miR-34a *(e.g. due to *p53 *mutations) may be a selective advantage in malignant cells.

In 5q- patients, we detected increased expressions of *miR-451 *and *miR-223 *(p < 0.01 and p = 0.0503 after Bonferroni correction) involved in the regulation of erythropoiesis that is defective in 5q- syndrome. *miR-451 *is a positive regulator of erythroid cell maturation. Its expression is very low at early stages of erythroid differentiation and rapidly increases with maturation progress [10]. However, the tested progenitors of 5q- patients displayed significant overexpression of *miR-451*. Target gene(s) of human *miR-451 *are still unclear. In zebrafish, *miR-451 *transcription is activated by a hematopoietic transcription factor GATA-1 and it likely controls erythropoiesis via *GATA-2 *target gene [11].

*miR-223 *is a transcriptional target of *CEBPA *and plays an essential role in granulopoiesis in which targets *E2F1 *[12]. In erythropoiesis, ectopic expression of *miR-223 *suppresses protein levels of LMO2 and thus impairs cell differentiation. Further, hematopoietic progenitor cells transduced with *miR-223 *show a significant reduction of their erythroid clonogenic capacity [13]. It suggests that down-modulation of this miRNA is required for erythroid progenitor recruitment and commitment. We may speculate that the overexpression of *miR-451 *and *miR-223 *interferes with the erythroid differentiation in 5q- syndrome.

In total BM cells, Hussein et al. detected increased transcript levels of *miR-199a*, *miR-125a*, and *miR-125b *in MDS patients with associated del(5q) [4]. We observed the same expression pattern of these miRNAs in 5q- BM progenitors. *miR-125a *and *miR-125b *are members of a multigene family located in paralogous clusters. The *miR-125a *cluster on chromosome 19 includes *miR-99b *and *let-7e*, whereas the *miR-125b *cluster on chromosome 21 consists of *miR-99a *and *let-7c*. We might conclude that these clusters were up-regulated in 5q- progenitors since *miR-125a*, *miR-125b*, *miR-99b*, *miR-99a *and *let-7e *showed increased levels before post p-value correction. Strong up-regulation of *miR-125b *is also found in MDS and AML with t(2;11)(p21;q23) and *in vitro *studies show its interference with primary human CD34+ cell differentiation [14]. In leukemic cell lines, *miR-125b *inhibits monocytic and granulocytic differentiation [14]. *miR-125a *is preferentially expressed in long-term hematopoietic stem cells and its activity is associated with induction of stem cell expansion [15].

Expression of *miR-128b *showed significant decrease in our 5q- patients. In contrast, it is overexpressed in acute leukemias and represents one of the most discriminatory miRNAs for ALL and AML [16]. Interestingly, *miR-128b *is down-regulated in *MLL*-rearranged ALL and targets *MLL *and *AF4 *genes involved in the fusion (including their fused variants) [17]. Both these target genes play an important role in leukemogenesis. Further, we detected down-regulation of *miR-342 *expression in 5q- patients. This miRNA likely plays a positive regulatory role in the granulocytic differentiation as demonstrated in acute promyelocytic leukemia treated by all-trans-retinoic acid [18].

**Figure 2:**
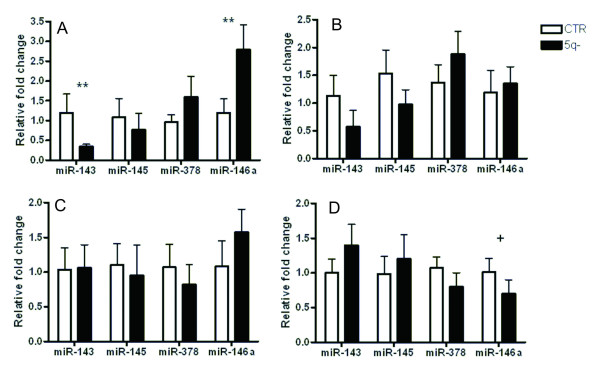
**Gene expression of miR-143, miR-145, miR-378 and miR-146a mapped within the del(5q) in 5q- patients**. Transcript levels of the miRNAs were detected by qRT-PCR in control (N = 12) and 5q- patient (N = 19) **peripheral blood granulocytes (A)**, **T-lymphocytes (B), monocytes (C)**, and **bone marrow CD34+ cells **(N = 7) **(D)**. Relative fold changes of expression were calculated by 2^-ΔΔCt ^and the data are presented as the mean plus standard error. Data were normalized to *RNU48*. CTR-controls, + p = 0.05, ** p < 0.01.

We attempted to address a gene dosage effect of del(5q) on expression of the miRNAs mapped within this deletion. Using a singleplex qRT-PCR, we analyzed gene expression of *miR-143*, *miR-145*, *miR-378 *and *miR-146a *in peripheral blood (PB) granulocytes, monocytes, and T-lymphocytes of 5q- patients and controls and in BM CD34+ cells used for miRNA expression profiling [Figure 2]. The miRNAs showed similar gene expression patterns in various PB cells. However, the pattern in BM progenitors was considerably different, demonstrating tissue-specific regulation of these miRNAs. In PB cells, *miR-143 *and *miR-145 *were expressed at the lower levels in patient granulocytes (*miR-143 *at p < 0.01) and T-lymphocytes. In patient monocytes, both miRNAs were expressed at the control level. *miR-378 *showed non-significant up-regulation in patient granulocytes and T-lymphocytes. *miR-146a *was up-regulated in all PB patient cells and reached statistical significance in granulocytes (p < 0.01). In BM CD34+ patient cells, *miR-143 *and *miR-145 *expressions showed slight up-regulation and transcript levels of *miR-378 *and *miR-146a *(p = 0.05) were reduced. Similar transcript levels of these miRNAs in BM CD34+ cells of 5q- patients were found by others [19]. Moreover, Boultwood et al. determined similar expression pattern of these miRNAs in BM CD34+ cells of refractory anemia patients with normal karyotype, demonstrating their 5q- syndrome non-specific regulation. In contradiction with these findings, Straczynowski et al. detected significant down-regulation of *miR-143 *and *miR-145 *in 5q- marrow cells and down-regulation of *miR-145 *and *miR-146a *in CD34+ cells of three 5q- patients [20]. In mouse, they further showed that knock-down of *miR-145 *and *miR-146a *caused thrombocytosis, neutropenia and megakaryocytic dysplasia [20]. The result discrepancy might arise from different numbers of tested patients and/or different clinical parameters of patients in the cohort (e.g. number of patients with thrombocytosis). Collectively, our results demonstrate that gene expression of the miRNAs in the deleted region is not significantly affected (resp. reduced) by the loss of one allele. We assume that there are mechanisms for compensation of the gene dosage (e.g. epigenetic modification causing higher expression of the retained allele) and the miRNAs cooperate to induce 5q- phenotype.

Target genes of some miRNAs at del(5q) were determined by functional studies. *TRAF6 *is target gene of *miR-146. miR-145 *targets *TIRAP *that is upstream of *TRAF6 *in Toll-like receptor pathway [20]. Induced expression of *TRAF6 *or knock-down of *miR-145 *and *miR-146a *in mouse BM cells leads to the 5q- like phenotype [20]. In our study, *miR-146a *expression was down-regulated by 30% in BM CD34+ cells of 5q- patients. Interestingly, gene expression of this miRNA showed increased levels in PB patient cells. Pons et al. detected also overexpression of *miR-146 *in BM mononuclear cells of MDS patients with del(5q) [3].

To define potential downstream targets of the deregulated miRNAs in 5q- patients, we performed an integrative analysis of differently expressed mRNAs and *in silico *predicted targets of the miRNAs. Messenger RNA profiles of CD34+ cells of the tested subjects have been previously assayed using Illumina HumanRef-8 v2 Expression BeadChips with 22,000 gene probes. Comparative analysis of 5q- patients and controls detected 246 differently expressed mRNAs (p < 0.01) [5]. We observed an inverse association between expressions of these miRNAs and targets; e.g. *miR-199a *and *CCDC34, MRPL22*; *miR-34a *and *LEF1*, *KLF4*, *SPI1*, *NR4A2*. All results are summarized in the Table 1.

**Table 1:** Potential target genes of deregulated miRNAs in 5q- patients.

miRNA ID	Location	Expression in 5q-	Target genes with inverse expression
**hsa-miR-34a**	1p36.22	over	LEF1, KLF4, SPI1, NR4A2
**hsa-miR-223**	Xq12	over	EBF3, PDE4B, PLEKHM1, RHOB
**hsa-miR-199a**	19p13.2	over	CCDC34, MRPL22
**hsa-miR-335**	7q32.2	over	ETF1
**hsa-miR-128b**	3p22.3	down	GPAM, RGL2
**hsa-miR-520c**	19q13.42	down	CYBRD1

Messenger RNA profiles of CD34+ cells in the tested subjects were assayed by microarrays and putative targets of deregulated miRNAs (p ≤ 0.05) were determined by prediction algorithm tools. The integrative analysis of differently expressed mRNAs and predicted targets identified genes with an inverse expression pattern.

As shown in the target list, we noted several potential target genes (e.g. *KLF4*, *LEF1*, *SPI1*) with an important function in the regulation of hematopoiesis. *KLF4 *gene is a member of the Kruppel-like family of factors (KLFs) that play essential roles in erythrocyte and lymphocyte development. *KLF4 *is expressed in a stage-specific pattern during myelopoiesis and its forced expression in hematopoietic stem cells promotes monocyte differentiation [21]. *KLF4 *is targeted by transcriptional factor PU.1 (SPI1) that cooperates with CEBPA to control myeloid cell development. LEF1 is a lymphoid enhancer-binding factor which mediates proliferation, survival and differentiation of granulocyte progenitors. Reduction/deficiency of *LEF1 *gene leads to the defective maturation of myeloid progenitors in patients with severe congenital neutropenia [22]. The down-regulation of these targets in 5q- patients may be associated with impaired cell differentiation. Taken together, our data underline complexity of the miRNA/target regulatory network that is being analyzed in detail by functional studies.

Our study demonstrates that the specific miRNA signature (mostly miRNA overexpressions) is associated with 5q- phenotype. Especially, *miR-34a *was highly up-regulated in 5q- syndrome patients. Notably, we found the up-regulation of several miRNAs (*miR-10a/b*, *miR-126*, *miR-99b*, and *miR-130a*) implicated in the regulation of *HOX *genes. Against our expectation, not all miRNAs at del(5q) displayed reduced gene expression. The analysis of mRNA expressions and predicted targets showed that aberrantly expressed miRNAs might be involved in the pathogenesis of 5q- syndrome by the modulation of their target genes.

## Methods

### Samples

Bone marrow (BM) CD34+ cells were obtained from 7 patients and 5 controls. Control CD34+ cells were purchased from Lonza (Basel, Switzerland). Peripheral blood (PB) granulocytes, T-lymphocytes and monocytes were obtained from 19 patients and 12 controls. All patients fulfilled WHO diagnostic criteria of 5q- syndrome. All subjects provided the informed consent and the study was approved by the Local Ethics Committee.

Mononuclear cells and granulocytes were separated by Ficoll-Hipaque density gradient centrifugation (GE Health Care, Little Chalfont, UK). Other cell fractions were isolated by magnetic column separation using MACS kits (Myltenyi Biotech, Bergisch Gladbach, Germany): Direct CD34+ Progenitor Cell Isolation Kit, CD3 T-Cells Isolation Kit, and CD14 Monocytes Isolation Kit. The purity of cell populations was controlled using FACSAria (Becton Dickinson, San Jose, CA) and always exceeded 95%.

### Analysis of miRNA expression

Total RNA (800 ng) was reverse transcribed into cDNA by TaqMan MicroRNA Reverse Transcription Kit (Applied Biosystems, Foster City, CA). TaqMan Human MicroRNA Arrays v1.0 (Applied Biosystems) with 365 probes were analyzed on 7900HT Fast Real-Time PCR System (Applied Biosystems). The array data were processed using SDS v2.3 (Applied Biosystems) and Genesis 1.6.0Beta1 software http://genome.tugraz.at/. Unsupervised hierarchical clustering of the data was done by average linkage and Euclidean distance. Singleplex qRT-PCR of *miR-143*, *miR-145*, *miR-378 *and *miR-146a *was performed on RotorGene 3000 instrument (Qiagen, Hilden, Germany) using TaqMan MicroRNA Expression Assays (Applied Biosystems). The PCR reactions were performed in duplicates. Relative gene expressions of these four miRNAs correlated (r = 0.97, p < 0.01) to those detected by multiplex qRT-PCR Array in bone marrow samples.

All miRNA data were normalized to the endogenous control *RNU48 *and relative fold changes of gene expression were calculated by ΔΔC_T _method. The statistical significance between miRNA expression of patients and controls was calculated by Student's t-test. The p-values were adjusted by Bonferroni correction for multiple testing.

Putative target genes were predicted using algorithm tools TargetScan 5.1 http://www.targetscan.org and PicTar http://pictar.mdc-berlin.de.

## Competing interests

The authors declare that they have no competing interests.

## Authors' contributions

HV, MG, MB and AV performed the experimental work and organized data. MDM analyzed array data. HV interpreted data and drafted the manuscript. RN and JC critically reviewed the manuscript and provided concepts. All authors read and approved the final manuscript.

## Supplementary Material

Additional file 1 **Fold changes of miRNA expressions in 5q- patients detected by TaqMan MicroRNA Arrays**. The data are presented as ratio of averaged expression in 5q- patients to averaged expression in controls for particular miRNAs. Only fold changes with p < 0.05 after Bonferroni correction are shown. Click here for file
