# Supplementary material for: Differential Expression of MicroRNAs in CD34+ Cells of 5q- Syndrome
Source: J Hematol Oncol. 2011 Jan 6;4:1. doi: 10.1186/1756-8722-4-1 (PMC3024999; doi:10.1186/1756-8722-4-1)
Supplement: Additional file 1 — Fold changes of miRNA expressions in 5q- patients detected by TaqMan MicroRNA Arrays. The data are presented as ratio of averaged expression in 5q- patients to averaged expression in controls for particular miRNAs. Only fold changes with p < 0.05 after Bonferroni correction are shown. [file 1756-8722-4-1-S1.DOC]

**Additional file 1**

**Fold changes of miRNA expressions in 5q- patients detected by TaqMan MicroRNA Arrays.** The data are presented as ratio of averaged expression in 5q- patients to averaged expression in controls for particular miRNAs. Only fold changes with p<0.05 after Bonferroni correction are shown.

| **miRNA ID** | **Accession**  **Mature sequence** | **Fold change** |
| --- | --- | --- |
| hsa-miR-10a | MIMAT0000253 | 2.43 |
| hsa-miR-10b | MIMAT0000254 | 2.06 |
| hsa-miR-125a | MIMAT0000443 | 2.70 |
| hsa-miR-125b | MIMAT0000423 | 1.89 |
| hsa-miR-126 | MIMAT0000445 | 1.78 |
| hsa-miR-128b | MIMAT0000424 | 0.21 |
| hsa-miR-130a | MIMAT0000425 | 1.39 |
| hsa-miR-148a | MIMAT0000243 | 2.65 |
| hsa-miR-151 | MIMAT0004697 | 1.80 |
| hsa-miR-199a | MIMAT0000231 | 2.10 |
| hsa-miR-199b | MIMAT0000263 | 2.58 |
| hsa-miR-213 | MIMAT0000256 | 0.48 |
| hsa-miR-24 | MIMAT0000080 | 1.59 |
| hsa-miR-29c | MIMAT0000681 | 1.81 |
| hsa-miR-335 | MIMAT0000765 | 1.85 |
| hsa-miR-34a | MIMAT0000255 | 12.68 |
| hsa-miR-451 | MIMAT0001631 | 3.07 |
| hsa-miR-486 | MIMAT0002177 | 3.12 |
| hsa-miR-95 | MIMAT0000094 | 0.36 |
| hsa-miR-99b | MIMAT0000689 | 1.83 |
| hsa-miR-520c | MIMAT0005455 | 0.44 |
